# A framework and process for community-engaged, mixed-methods cancer needs assessments

**DOI:** 10.1007/s10552-024-01892-2

**Published:** 2024-05-29

**Authors:** Todd Burus, Jessica R. Thompson, Caree R. McAfee, Lovoria B. Williams, Jennifer Redmond Knight, Bin Huang, Sarojini Kanotra, Natalie P. Wilhite, Elaine Russell, Melinda Rogers, Connie L. Sorrell, Christine Stroebel, Rachael King, Pamela C. Hull

**Affiliations:** 1 Markey Cancer Center, University of Kentucky, Lexington, KY USA; 2 College of Nursing, University of Kentucky, Lexington, KY USA; 3 Kentucky Cancer Consortium, Lexington, KY USA; 4 Department of Health Management and Policy, College of Public Health, University of Kentucky, Lexington, KY USA; 5 Division of Cancer Biostatistics, College of Medicine, University of Kentucky, Lexington, KY USA; 6 Kentucky Cancer Registry, Markey Cancer Center, University of Kentucky, Lexington, KY USA; 7 Kentucky Department for Public Health, Frankfort, KY USA; 8 Kentucky Cancer Program, Markey Cancer Center, University of Kentucky, Lexington, KY USA; 9 Kentucky Cancer Program, Brown Cancer Center, University of Louisville, Louisville, KY USA; 10 American Cancer Society, Kennesaw, GA USA; 11 Department of Behavioral Science, College of Medicine, University of Kentucky, Lexington, KY USA; 12 University of Kentucky Markey Cancer Center, 760 Press Avenue, Suite 460, Lexington, KY 40536 USA

**Keywords:** Catchment area, Needs assessment, Qualitative research, Quantitative evaluation, Community outreach

## Abstract

**Purpose:**

Community health needs assessments are required for most state and local public health agencies and non-profit hospitals. Typically based on community health improvement planning models, these assessments encompass overall community health and multiple diseases to inform program planning. National Cancer Institute (NCI)-designated Cancer Centers and community-based cancer-focused programs share the goal of reducing cancer burden in the catchment areas they serve. However, to date, no published models exist to guide cancer-specific needs assessments for a determined geographic area that can inform both public health and research initiatives. The purpose of this article is to outline a cancer needs assessment (CNA) framework and community-engaged, mixed-methods process, along with a case study of how we applied it in Kentucky.

**Methods:**

We convened a steering committee of key organizational partners to provide input throughout the process. We developed a conceptual framework of multi-level determinants affecting cancer-related outcomes. We incorporated both quantitative and qualitative data gathered through a variety of means, including a novel application of group concept mapping to guide definition of priorities.

**Results:**

The resulting CNA has helped guide strategic planning and priorities for Kentucky’s Cancer Action Plan, Markey Cancer Center, state agencies, and community-based organizations.

**Conclusion:**

This framework and process can be used collaboratively by cancer center Community Outreach and Engagement offices, public health agencies, oncology programs, and community partners to plan impactful cancer control programs and research in their catchment areas. Universities can also use them to inform the planning of community engagement and health equity research efforts.

**Supplementary Information:**

The online version contains supplementary material available at 10.1007/s10552-024-01892-2.

## Introduction

Needs assessments arose in the mid-twentieth century as a useful tool for performing program planning and evaluation. Their purpose is to, first, identify needs (the gap between the current and future desired conditions) and then prioritize how to address them [1]. State and local public health agencies were early adopters of community health needs assessments (CHNA), and since 2011, the national Public Health Accreditation Board has required CHNAs [2]. In recent years, needs assessments have taken a more prominent role in healthcare with the requirement that nonprofit hospitals perform a CHNA every 3 years in accordance with the Patient Protection and Affordable Care Act (ACA) of 2010 [3]. Hospitals are also required to develop a corresponding implementation plan to act on priorities identified through their CHNAs. Combined, this process is meant as an accountability measure for facilities receiving federal funding, pushing them to focus on health outcome improvements for individuals living within their service areas. In 2012 the Commission on Cancer (CoC) under the American College of Surgeons initiated new requirements for CoC-accredited oncology treatment facilities to perform a CHNA once every 3 years [4]. While the 2020 CoC standards no longer require CHNAs, their implementation is encouraged to guide outreach and psychosocial programs that address barriers to cancer care [5].

To promote local accountability and community benefit, in 2013, the National Cancer Institute (NCI) introduced the concept of catchment areas in the Cancer Center Support Grant (CCSG) funding requirements for NCI-Designated Cancer Centers. A cancer center catchment area is a population- or geographically based area in which the cancer center does or desires to serve patients and perform research to reduce the cancer burden [6]. The subsequent 2016 CCSG guidelines expanded on prior requirements for cancer prevention and control by establishing a new Community Outreach and Engagement (COE) component [7]. The COE component was charged with continuously generating a comprehensive profile of the catchment area’s unique cancer needs (i.e., factors influencing poor cancer-related health outcomes and disparities) and opportunities for improving them. This profile should catalyze both cancer control activities and basic, clinical, translational, and population research priorities to address identified needs in collaboration with community partners [8]. Conducting a needs assessment can help cancer center COE offices accomplish these objectives [9, 10].

Public health agencies typically follow CHNA processes outlined by community health improvement planning (CHIP) models, which emphasize involving local residents and partner organizations to assess community health status; identify health priorities; and plan, implement, and evaluate city- or statewide health improvement initiatives [11–13]. In contrast, the non-profit hospital CHNA requirement did not come with much guidance and, as such, has produced varied results during its first decade of existence [14]. Furthermore, CHIP-based CHNA processes are designed to encompass the entire spectrum of community health and select priorities among multiple diseases, with a narrow focus on planning public health agency programs. Additionally, the National Comprehensive Cancer Control Program requires funded states, tribes and territories and their cancer coalitions to review cancer-related data and develop a jurisdiction-wide cancer plan [15]. No formal process is in place to fulfill this requirement, and it varies considerably among states and jurisdictions [16]. To date, no published models exist to guide cancer-specific needs assessments that can inform public health initiatives, cancer plans, and research.

To fill this gap, the objectives of this article are to: (1) present a cancer needs assessment (CNA) framework and community-engaged, mixed-methods process to guide the identification of priorities for both community-based cancer control activities and research agendas aimed at reducing cancer burden and disparities in a specific catchment area, and (2) illustrate a case study of how we applied the CNA framework and process in Kentucky through a partnership among the University of Kentucky Markey Cancer Center (UKMCC) COE team and a steering committee comprised of academic, public health, and community partners. While separate articles will detail the methods and results of specific components of the Kentucky CNA in greater depth, this article provides a high-level overview of how to conduct a community-engaged, multi-method CNA that integrates various sources and types of data.

## Methods

### Conceptual framework

We drew on several existing models to develop a conceptual framework of social and individual factors that influence cancer outcomes to guide the collection, analysis, interpretation, and presentation of data in Cancer Needs Assessments (CNA). For example, Rodriguez et al. adapted McLeroy’s Social-Ecological Model and illustrated how nested interactions at the patient, community, and policy levels impact patient health outcomes and health disparities [17]. Hiatt and Breen went a step further by considering the direct influence different levels of analysis, including social determinants of health (SDOH), have on the cancer care continuum, and where opportunities for intervention exist [18, 19]. Additionally, the American Association for Cancer Research put forward a model which recognized the roles both personal and population evidence-based actions can play in effective cancer prevention [20]. Finally, Alcaraz et al. created a framework for advancing cancer health equity through understanding and addressing SDOH [21, 22]. The Alcaraz et al. model introduced the idea of an upstream/downstream intervention orientation in which they argue that “[to] achieve cancer health equity, more focused efforts are needed upstream to address social factors for population-level impact.”

**Fig. 1:**
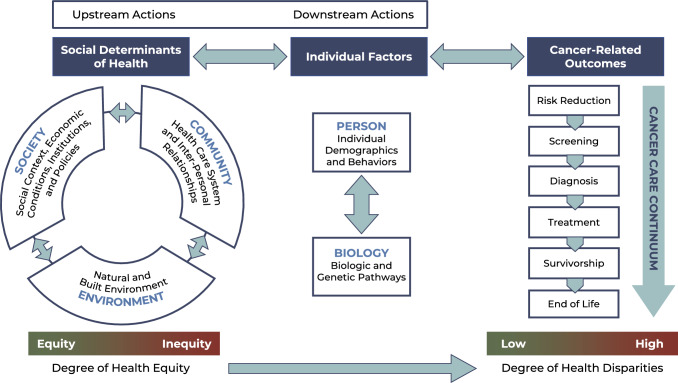
Multilevel determinants of cancer-related outcomes framework. Society includes social context (e.g., culture; social norms; the meaning of socially constructed concepts like race, ethnicity, and gender), economic conditions (e.g., median income, unemployment), institutions (e.g., education system, criminal justice system), and policies (e.g., Affordable Care Act, Medicaid, institutional policies). Environment includes the “natural environment” (e.g., water, air, soil) and substances (natural or man-made) that individuals are exposed to through these means, and the “built” environment constructed by humans (e.g., roads, sidewalks, parks, buildings). Community includes the local health care system (e.g., primary care, hospitals, cancer clinics, and other facilities) and interpersonal relationships (e.g., family, neighborhoods)

The UKMCC COE synthesized these concepts to develop a draft CNA conceptual framework, the Multilevel Determinants of Cancer-related Outcomes Framework, and the steering committee gave input to refine it (Fig. 1). This framework organizes several intertwined levels of consideration for assessing cancer needs and identifying potential interventions to reduce cancer burden and disparities. On the left, the SDOH reflect the overall context in which an individual lives. We classified SDOH as falling into three levels—society, environment, and community (see Fig. 1), which influence one another in a complex, circular manner over time [23]. In the middle, under individual-level factors, one’s personal characteristics and behaviors interact with their body’s internal biological mechanisms, which could potentially create somatic mutations and result in cancer. The complex interplay of contextual SDOH factors and individual factors interact with cancer-related outcomes along the cancer care continuum, shown on the right. On the bottom left, the green to red continuum indicates varying degrees of health equity in contextual SDOH factors for a given geographic area, which influence the risk of exposures and behaviors for individuals in different social groups (e.g., gender, race/ethnicity, rural/urban). The degree of health equity in SDOH impacts the degree of health disparities in population-level cancer-related outcomes across social groups, as indicated on the bottom right. Along the top of the figure, we indicate the continuum of upstream actions directed toward SDOH to downstream actions targeting the individual level.

### Cancer needs assessment mixed method process

**Fig. 2:**
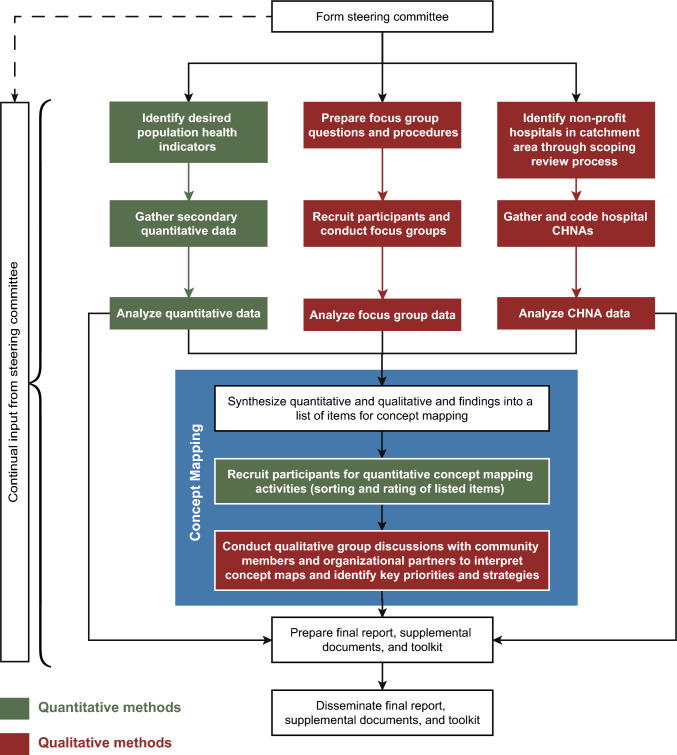
Process workflow for performing a community-engaged, mixed-methods cancer needs assessment

Mixed methods research brings together the strengths of quantitative breadth and qualitative depth of data within the same study to gain greater insights [24]. In needs assessments, mixed methods provide a comprehensive picture of community health needs and issues, adding lived experience to identified quantitative patterns [25]. Considering the availability of existing cancer-related quantitative data and important gaps that need to be filled with qualitative data, we recommend the process illustrated in Fig. 2, which represents a community-engaged, explanatory sequential mixed methods approach. The first phase of the CNA process includes assessment of patterns in existing quantitative data plus simultaneous gathering of qualitative community insights through focus groups and review of hospital CHNAs. While extensive secondary quantitative data exists in publicly available sources, groups may also choose to perform additional primary quantitative data collection (e.g., population health surveys), if they have sufficient financial and personnel resources [8, 26]. The second phase is a group concept mapping process with quantitative and qualitative steps to generate consensus on priorities. The final phase involves summarizing and disseminating CNA findings. A CNA steering committee of key stakeholders should ideally provide input throughout all phases. Below we describe the methodology of each component and illustrate the case study of how we applied this process for the Kentucky CNA (KY CNA).

### Community partner input

An essential preliminary step for a community-engaged CNA process is identifying a collaborative steering committee, which could be a new or existing committee, task force, or community advisory board. This group is typically comprised of key stakeholders within the catchment area representing a variety of points of view, such as local non-profit organizations, government agencies, universities, healthcare providers, policy makers, and other community leaders or members [25]. The committee’s responsibility is to provide input throughout the CNA process on design, recruitment, data collection, interpretation of findings, and dissemination. This collaborative approach grounds the CNA process in existing community insights and assets, ensures representation, and builds credibility and investment in the results among stakeholder organizations.

Given that the catchment area for the 2021 KY CNA encompassed the entire state, the UKMCC COE formed a KY CNA steering committee consisting of organizations and programs with a statewide reach, a focus that includes cancer, access to data sources or networks of organizations and community members, and a common interest in a statewide CNA. The 27-member steering committee included representatives of the UKMCC, Kentucky Cancer Consortium (KCC, the state cancer coalition), Kentucky Cancer Program (KCP, a statewide outreach program), Kentucky Cancer Registry (KCR), Kentucky Department for Public Health, American Cancer Society, Foundation for a Healthy Kentucky, and University of Louisville. From March 2021 to March 2022, through all steps of the process, UKMCC COE convened monthly steering committee meetings and also sought their input through email. In addition, UKMCC COE regularly updated and gathered feedback during the process from the UKMCC Community Advisory Board, comprised of 12 lay community members and community organizations.

### Quantitative methods

In performing a CNA, the quantitative methods provide a numerical view of the target catchment area. This assists in, among other things, measuring the observed degree of cancer burden, inequities in SDOH, and disparities in cancer-related outcomes that exists among certain population subgroups. For the 2021 KY CNA we chose to only gather secondary quantitative data due to practical considerations surrounding the large size of our catchment area, the ongoing COVID-19 pandemic at the time, and to focus available resources on gathering needed qualitative data.

#### Gathering secondary data

While developing the conceptual framework for the KY CNA, we began to brainstorm available quantitative data from secondary sources that correspond to components of the framework. We collected these ideas in an indicator list with notes about available sources and the additional demographic information each contained. Subsequently, we organized indicators based on the conceptual framework. Throughout the needs assessment process, we regularly revisited this list. By the end, this list contained over 100 quantitative variables to consider beyond just cancer incidence and mortality (Suppl. Table S1). We compiled this data, developed summaries, and analyzed it for the presence of significant disparities between various subgroups of the population. In a parallel project, the UKMCC COE streamlined the process of gathering of multiple publicly available data sources through the creation of a catchment area data collection software called Cancer InFocus [26].

#### Geographic levels

Given that the scope of our needs assessment was the entire state, we processed all of the data collected at the state, county, and/or census tract level (as available) to aid in creating geographic visualizations. Kentucky has a unique level of administrative boundaries utilized in this process known as Area Development Districts (ADDs). ADDs combine several counties into larger sub-state regions, making it possible to report on certain variables that would otherwise need to be suppressed at the smaller county level. Depending on the makeup of the area being assessed, it may be useful to collect available data for other geographic levels (such as public use microdata areas) or non-standard administrative regions.

#### Visualizations

We constructed numerous tables, charts, and maps with the quantitative data we collected. We shared these visualizations with our steering committee during monthly meetings to get feedback on ease of comprehension and how to best capture the story being told by the data. Consistent colors were used across visualizations to assist in developing a visual narrative that was easy to follow with minimal need for written text in the final document.

#### Dissemination follow-up survey

To evaluate the reach and impact of our CNA, a follow-up survey was sent to individuals who volunteered to be recontacted when downloading a digital copy of resources from the 2021 KY CNA website (www.kycancerneeds.org). The survey was constructed in REDCap and consisted of multiple questions intended to assess the usage of needs assessment findings throughout the state [27].

#### Data processing

Data processing was performed in the R statistical programming language, and visualizations were created in R (version 4.2.1, R Core Team, Vienna, Austria), Tableau (version 2021.3, Tableau, Seattle, WA), and ArcGIS Pro (version 2.9, Environmental Systems Research Institute, Redlands, California). When formal statistical tests were performed to compare values, a *p*-value of 0.05 was used to assess statistical significance. Otherwise, statistically significant differences were noted using non-overlapping 95% confidence intervals.

### Qualitative methods

The qualitative aspect of a mixed methods CNA allows researchers to examine the perceived needs in the community and add additional context to the inequities and disparities observed using quantitative methods. Importantly, qualitative methods allow for the inclusion of voices in needs assessments from underrepresented portions of the population whose experiences may not be captured by solely relying on quantitative population-level data [25]. Additionally, through open-ended questions, qualitative data can provide details on the ‘how’ and ‘why’ certain quantitative trends exist in order to develop the community-specific strategies necessary to address identified needs [24].

#### Hospital community health needs assessments scoping review

We conducted a scoping review of hospital CHNAs from all non-profit hospitals across the state of Kentucky to improve our understanding of statewide priority health needs and implementation strategies and to identify where priority needs aligned with the cancer care continuum. The CHNA review process included: (1) Conducting a literature review of the CHNA process; (2) Collecting the most recent CHNA and implementation strategy reports for Kentucky hospitals; (3) Documenting information about hospital catchment areas from CHNAs and building hospital profiles; (4) Building a glossary of terms and definitions, and grouping them into categories under priority health needs and implementation strategies; (5) Training staff on how to review and code priority health needs and implementation; (6) Performing two reviews per CHNA; and (7) Reconciling discrepancies in categorization between initial reviews with a third reviewer.

#### Focus groups

To incorporate more community participation beyond our steering committee, we conducted online focus groups with adult (age 18+) residents of Kentucky who did not work in a healthcare profession (*n* = 51). From July to September 2021, we recruited participants utilizing our existing KCC and KCP partnership networks and ResearchMatch, an online volunteer research registry [28]. Our study team contacted eligible participants, gathered study consent, and assigned participants to a specific scheduled focus group. As we sought to identify needs by race and ethnicity, geographic area, and sexual orientation and gender identity, we intentionally stratified groups by these characteristics based on a brief demographic questionnaire, resulting in 11 focus group sessions.

The focus group discussions lasted approximately two hours and took place via Zoom. Discussions were led by a trained facilitator and supported by a research assistant. Our semi-structured questions focused on participant awareness of existing resources and needs across the cancer care continuum. We supported this discussion using a visual graphic displaying the various areas of the cancer care continuum (risk reduction, screening, treatment, and follow-up/survivorship). Participants received a $50 e-gift card for their participation. Each focus group discussion was recorded and transcribed for qualitative coding analysis. The facilitator and research assistant conducted a line-by-line review of the transcripts and double-coded 20% of the transcripts, resulting in over 90% agreement. Any discrepancies were resolved through consensus.

### Concept mapping

After focus group data collection, we designed a concept mapping project to prioritize the wide variety of identified cancer needs and strategies. A participatory mixed method, concept mapping utilizes a series of survey-based activities and group discussions to generate consensus on a particular topic of interest [29–31]. From September to December 2021, we recruited community members (adults, non-health professionals, and Kentucky residents) and community organization employees who work in cancer services in Kentucky to participate in concept mapping remotely (*n* = 162). The community member participants were individuals who previously participated in the KY CNA focus groups. Community organizations were identified by KCC and KCP and included representatives from health departments, nonprofit organizations, advocacy groups, insurance companies, health systems, and educational organizations.

We recruited participants by email and provided a link to the online concept mapping activities utilizing the Groupwisdom concept mapping platform [32]. Prior to beginning the first set of activities, a working group of COE, KCP, and KCC members compiled a list of 80 items from the previous quantitative findings, qualitative focus group discussion themes, and concerns raised at partner meetings. The survey-based activities asked participants to sort these 80 items into thematic groups and to rate each item on two five-point Likert-type scales: (1) importance for Kentucky communities and (2) how easy it would be to address in Kentucky communities (i.e., feasibility). With these data, the study team generated concept maps (e.g., point and cluster maps) using cluster analysis and rating assessments (e.g., correlational comparisons), which were shown to participants in group discussions. We conducted six discussion groups via Zoom, including three with community members and three with organizational partners. The community member participants received up to $60 in e-gift cards for their participation ($30 for the online survey-based activities and $30 for the group discussion).

### Dissemination

We used a multi-pronged strategy for disseminating the KY CNA report and district profiles across Kentucky. This approach included distributing physical copies of the summary report and district profiles and creating a website with the digital summary report, district profiles, and interactive data dashboard. In addition, we hosted meetings and webinars on the findings of the report and how they could be used to inform research, guide strategic planning and outreach, and impact the community.

### Institutional review board statement

UK Institutional Review Board (IRB) approved the research procedures under three protocols: Kentucky Behavioral Risk Factor Survey analysis (#69894), focus groups (#65451), and concept mapping (#73420). The IRB issued a non-human research determination for using the publicly available, aggregated secondary data and the CNA dissemination follow-up survey.

## Results

### Quantitative results

We gathered secondary data from over 18 different sources including KCR, a population-based central cancer registry for the Commonwealth of Kentucky and member of the NCI Surveillance, Epidemiology, and End Results program since 2000 (Table 1). KCR provided cancer incidence and mortality data for 2014–2018 for all primary cancer sites stratified by combinations of sex, race, rurality, Appalachian residence, and county or ADD. They also calculated new combined incidence and mortality rates for three groups of cancer sites associated with the major cancer risk factors of tobacco, obesity, and human papillomavirus (HPV) [33–35]. Twelve of the top 20 incidence rate cancers, and 12 of the top 20 mortality rate cancers, were observed to have significantly higher rates in Kentucky than in the rest of the U.S. (Suppl. Table S2; Fig. S1). Of particular interest, we found that Kentucky lung cancer incidence rates were 78.7% higher than U.S. rates, and lung cancer mortality rates were 81.9% higher. Kentucky also experienced significantly higher rates in incidence and mortality for the groupings of tobacco-, obesity-, and HPV-related cancers.

**Table 1:** Data sources used in the 2021 Kentucky Cancer Needs Assessment

Domain	Source	Year(s) used
Social determinants of health	American Community Survey, U.S. Census Bureau	2015–2019
	Appalachian Regional Commission	2021
	County Health Rankings & Roadmaps, University of Wisconsin Population Health Institute	2021
	Department for Medicaid Services, KY Cabinet for Health and Family Services	2021
	Food Access Research Atlas, U.S. Department of Agriculture Economic Research Service	2015
	Form 477, Federal Communications Commission	2021
	Health Resources & Services Administration, Department of Health & Human Services (HHS)	2021
	National Flood Hazard Layer, Federal Emergency Management Agency	2021
	Toxic Release Inventory, U.S. Environmental Protection Agency	2021
Individual factors	Behavioral Risk Factor Surveillance System and Kentucky Behavioral Risk Factor Surveillance, Centers for Disease Control and Prevention (CDC) and Kentucky Department for Public Health	2016–2019
	National Immunization Survey-Teen, CDC	2015–2019
	National Notifiable Diseases Surveillance System, CDC	2021
	Youth Risk Behavior Surveillance System, CDC	2019
Cancer-related outcomes	Certified Mammography Facilities, U.S. Food & Drug Administration	2021
	Kentucky Cancer Registry	2014–2018
	Lung Cancer Screening Registry, American College of Radiology	2021
	National Plan and Provider Enumeration System, HHS	2021
	Surveillance, Epidemiology, and End Results Program, National Cancer Institute	2014–2018

Likewise, we noted significant disparities among several behavioral risk factors and socioeconomic factors, though Kentucky’s rates for cancer screening were comparable to U.S. rates (Suppl. Table S3; Fig. S2). KCR provided relative survival rates for cancer patients for the years of 2012–2018, defined as the percentage of patients with a particular cancer diagnosis surviving 5-years after being diagnosed compared to a similar population of individuals without a cancer diagnosis. This revealed that Black Kentucky females with a breast cancer diagnosis had a significant 10% lower 5-year survival rate than White Kentucky females.

### Qualitative results

#### Community health needs assessments scoping review

**Fig. 3:**
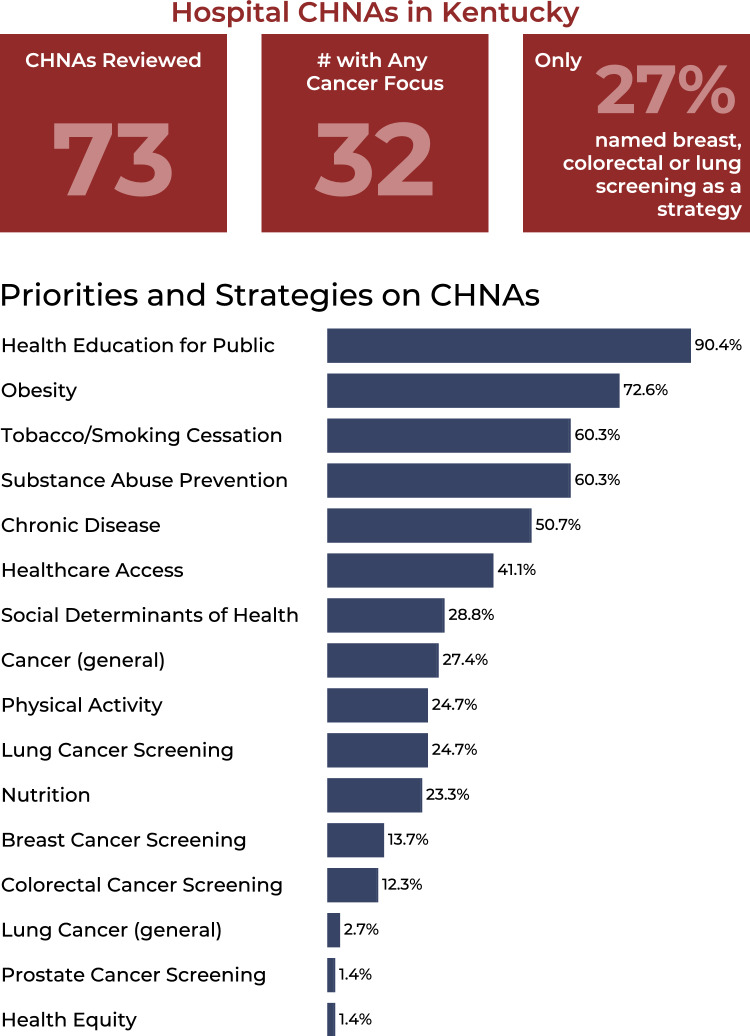
Bar chart showing the results of the Kentucky non-profit hospitals Community Health Needs Assessments (CHNA) review, indicating the percentage of CHNAs that include these items as a priority and/or strategy

One-hundred and ten non-profit hospitals in the state of Kentucky met the criteria for needing to have performed a CHNA at the time of our review. Research staff successfully located CHNAs for 73 of these hospitals from websites and requesting copies. We analyzed these CHNAs for content on their strategic priorities and implementation strategies (Fig. 3). While 60.3% of them noted tobacco/smoking cessation as a strategic priority, only about a quarter (27.4%) specifically highlighted cancer. Lung cancer screening was a priority for 24.7% of hospitals reviewed, breast cancer screening for 13.7%, and colorectal cancer screening for 12.3% (no hospital highlighted a priority for cervical cancer screening despite Kentucky ranking first among U.S. states in cervical cancer incidence). Mention of focusing on SDOHs appeared on 28.8% of CHNAs reviewed, while health equity was only found in 1 of the 73.

#### Focus groups

Using an online format, we uncovered a wide variety of factors affecting Kentuckians across the cancer care continuum. For example, continued novel approaches are needed to make information accessible and to utilize messaging that will not be interpreted as blaming or shaming. Likewise, screening efforts need to continue reaching individuals where they are and include messaging from individuals who engender trust. Continued efforts are also needed to address practical concerns for both screening and treatment, such as cost and transportation as well as lack of knowledge of which screening tools and treatments are covered by insurance. Participants expressed unique concerns based on their race and ethnicity, rurality, sexual orientation and gender identity, and age. These differences suggest uncovering ways to promote positive, understanding communication between patients and providers and to create safe care spaces that consider ways cultural norms affect cancer care to fight stigma and to improve health equity [36].

### Concept mapping results

**Fig. 4:**
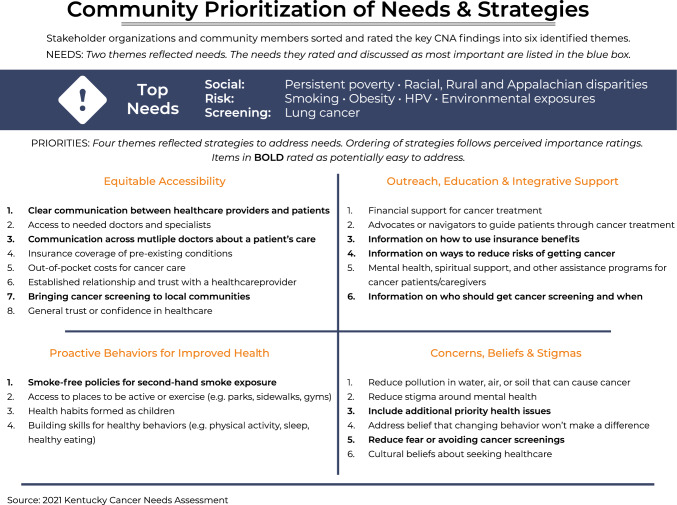
Community prioritization of needs and strategies from the 2021 Kentucky Cancer Needs Assessment

The concept mapping process successfully grouped the 80 items, which span factors across the cancer care continuum, into six thematic clusters. We also identified potential community-driven strategies to improve cancer risk reduction, treatment, and follow-up in Kentucky (Fig. 4). Specifically, participants commonly identified key areas for continued efforts, such as lung cancer screening, smoking cessation, HPV vaccination, and disparities driven by social determinants among rural, Appalachian, Black, and Hispanic Kentuckians. Community member and partner-driven strategies to affect these areas include a continued focus on health communication strategies, supports for treatment navigation, ways to overcome barriers to accessing care, and methods for increasing trust in patient–provider relationships. Moving forward, healthcare professionals dedicated to improving cancer in Kentucky can consider ways to build upon these strategies.

### Final synthesis

Results from the quantitative and qualitative data collection were synthesized into a final KY CNA report entitled, “2021 Kentucky Cancer Needs Assessment: The Story of Cancer in Kentucky.” This 59-page report was broken down into five sections, including an executive summary of findings. Emphasis was placed on presenting information in a visual format with minimal prose where possible. Additional one-page district profiles were created using data from the report for each of Kentucky’s 15 ADDs.

### Dissemination results

**Fig. 5:**
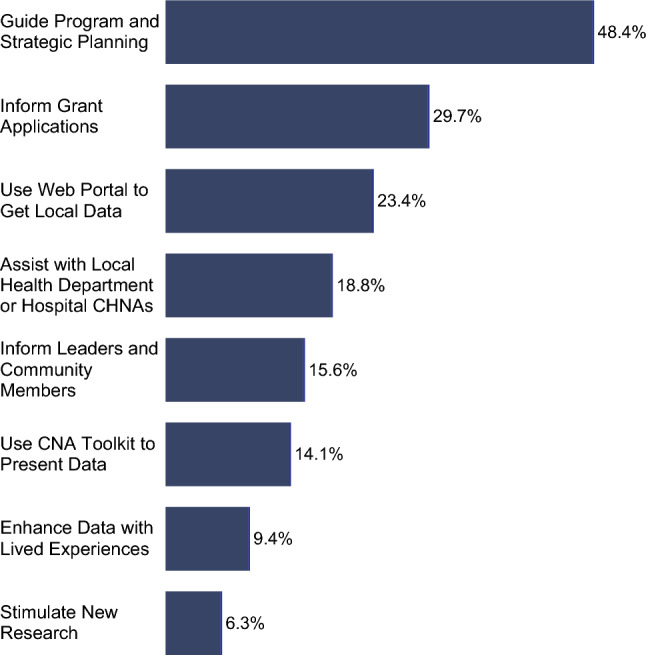
Bar chart showing how individuals used the 2021 Kentucky Cancer Needs Assessment (*n* = 64)

The KY CNA website (www.kycancerneeds.org) launched in April 2022 with a downloadable version of the summary report, along with two media toolkits breaking down the report visualizations into individual images for use in presentations and grant proposals. To facilitate tracking, we asked users to complete a request form to access the downloads, with the option of sharing contact information for a follow-up survey to be sent approximately 2 months later. Between April 2022 and January 2023, 304 people submitted the request form and 650+ report documents were downloaded. We sent 148 follow-up surveys. Among respondents (*n* = 64), the top uses of the CNA reported were to guide program and strategic planning (48.4%), to inform grant applications (29.7%), and using the web portal to get local data (23.4%) (Fig. 5). Use of the KY CNA was spread broadly across individuals in healthcare professions, university settings, state and local government, and cancer-focused non-profit organizations.

In addition, 750 physical copies of the KY CNA report were distributed throughout Kentucky, including 138 given to the current members of Kentucky’s state legislature. Over 2,000 copies of the district profiles were printed for dissemination by KCP Regional Cancer Control Specialists to local partners. Report findings were shared by UKMCC COE staff to over 800+ persons across 26 oral presentations.

## Discussion

We sought to outline a conceptual framework and process that cancer centers, cancer coalitions and other organizations can follow to perform community-engaged CNAs in their catchment areas. In particular, this project provided important methodological insight on how to triangulate quantitative and qualitative data and gather community feedback on setting research and outreach priorities. Applying this iterative process in the KY CNA, we were able to incorporate the perspectives of diverse community members across the state. Moreover, the contributions from focus groups, concept mapping, and our steering committee helped ensure that we could maximally assess the assets, opportunities, and barriers for cancer prevention and control. The KY CNA represented a significant advancement in understanding the story of cancer in Kentucky and laid the groundwork for future efforts to reduce the cancer burden in Kentucky.

Previous research has highlighted the importance of engaging community stakeholders in needs assessments and identification of priorities [12, 37]. However, in a review of CHNAs among Texas non-profit hospitals, Pennel et al. observed that 18% made no attempt to engage community members, and only four out of 76 involved community members in strategy selection [14]. Within the KY CNA steering committee, the Kentucky Cancer Program—which already operates within Kentucky communities—played an important role in facilitating the involvement of lay community members. These individuals represented key population subgroups in the state and provided crucial feedback in setting priorities and strategies—reflecting an “interpretive approach” to defining community as highlighted by Franz et al. [38]. Although a simple look at incidence and mortality rates reveals the disproportionate burden of cancer in Kentucky, the KY CNA findings illustrate that the causes of this problem and the avenues for addressing it require a much deeper analysis. This finding supports the argument of Pennel et al. that grappling with the broader social determinants of health is necessary to achieve a beneficial impact on cancer outcomes and the improvement of health equity [39].

Our CNA process also adds several methodological contributions to the identification and prioritization of catchment area needs through community-engaged and mixed method approaches. Specifically, our novel approach includes: (1) the use of virtual focus groups to engage a wide-range of community members throughout our catchment area, including oversampling populations most at risk (e.g., rural, Black, sexual, and gender minorities); (2) the employment of concept mapping with both community members and statewide organizational partners to prioritize the wide array of identified cancer needs in our catchment area; and (3) the combination of several quantitative and qualitative data sources to ultimately inform new strategic plans, both across our catchment area and for local healthcare systems and communities. The CNA results were subsequently used to inform a new Kentucky Cancer Action Plan. Our use of concept mapping provided an opportunity for resulting strategic plans to incorporate the practical considerations of local organizational partners and the lived experiences of those in the community. Although concept mapping has been utilized for program planning, evaluation, and community needs assessments previously, to our knowledge this is the first use of the approach by a cancer center in a cancer-focused needs assessment capturing needs in a statewide catchment area [31, 40]. Similar to other research conducted during the COVID-19 pandemic, we successfully adapted to pandemic restrictions by using virtual data collection for both the focus group and concept mapping elements [41, 42]. Our ability to capture perspectives from diverse lay community members in over 40 different Kentucky counties suggests that virtual qualitative data collection is a viable CNA method for cancer centers and other organizations seeking to broadly capture needs in diverse and/or large catchment areas.

Although this project makes important contributions to the literature, it contains certain limitations that we acknowledge. The available secondary quantitative data sources to assess cancer rates, social determinants of health, and behavioral risk factors have varying time delays. To moderate this impact, we used data from the most recent years available for each source. Additionally, our analysis is subject to all of the limitations that come with the available secondary sources—in particular, lack of information about sexual orientation, gender identity, and disabilities. Though collecting new (primary) survey data on individuals in our catchment area could have potentially contributed additional quantitative insights unavailable in the secondary sources, the large size of our catchment area and cost made doing so impractical. For focus groups and concept mapping, the steering committee expressed concerns regarding technological literacy among participants due to the use of virtual formats. We sought to address this issue by asking potential participants about their comfort level with the technologies used and, when requested, providing them trainings to increase participation. Our goal was to broadly capture diverse perspectives across the state, which we achieved. However, although we oversampled vulnerable populations, we did not include a sufficient number of participants from individual groups to form specific recommendations by demographic group. The qualitative methods used in this project were not intended to be generalizable, but were designed with the intent of gaining depth of understanding on needs and general consensus on priorities with respect to cancer specifically. Finally, we did not gather extensive process metrics beyond dissemination data to evaluate the process. However, the steering committee expressed satisfaction with the process throughout each phase. Future applications of this framework and process could gather additional process metrics to demonstrate efficacy.

## Conclusion

Performing periodic CNAs should be adopted as standard practice for cancer center COE offices and other organizations wishing to improve impact within their catchment area. The CNA framework aids in understanding the multilevel determinants of cancer-related outcomes to guide CNA planning and interpretation of findings. The CNA process employs a mixed-methods design with a variety of community input throughout to complement the quantitative data with people’s lived experiences. The resulting KY CNA report and dissemination not only informed the cancer center’s institutional research and strategic efforts, but also the efforts of state-level and community-based cancer organizations across Kentucky. This CNA framework and process can be replicated by COE offices at NCI-Designated Cancer Centers, public health agencies, cancer coalitions, oncology programs and community organizations. In addition, they can be used by universities for institution-level efforts focused on community engagement, health equity, and cancer research that spans across multiple research projects.

## Supplementary Information

Below is the link to the electronic supplementary material.

Supplementary file1 (PPTX 702 kb)

## Data Availability

Inquiries about data availability should be directed to the authors.
