# Supplementary material for: A framework and process for community-engaged, mixed-methods cancer needs assessments
Source: Cancer Causes Control. 2024 May 29;35(10):1319–32. doi: 10.1007/s10552-024-01892-2 (PMC11461567; doi:10.1007/s10552-024-01892-2)
Supplement: Supplementary file 1 — Supplementary file1 (PPTX 702 kb) [file 10552_2024_1892_MOESM1_ESM.pptx]

## Slide 1
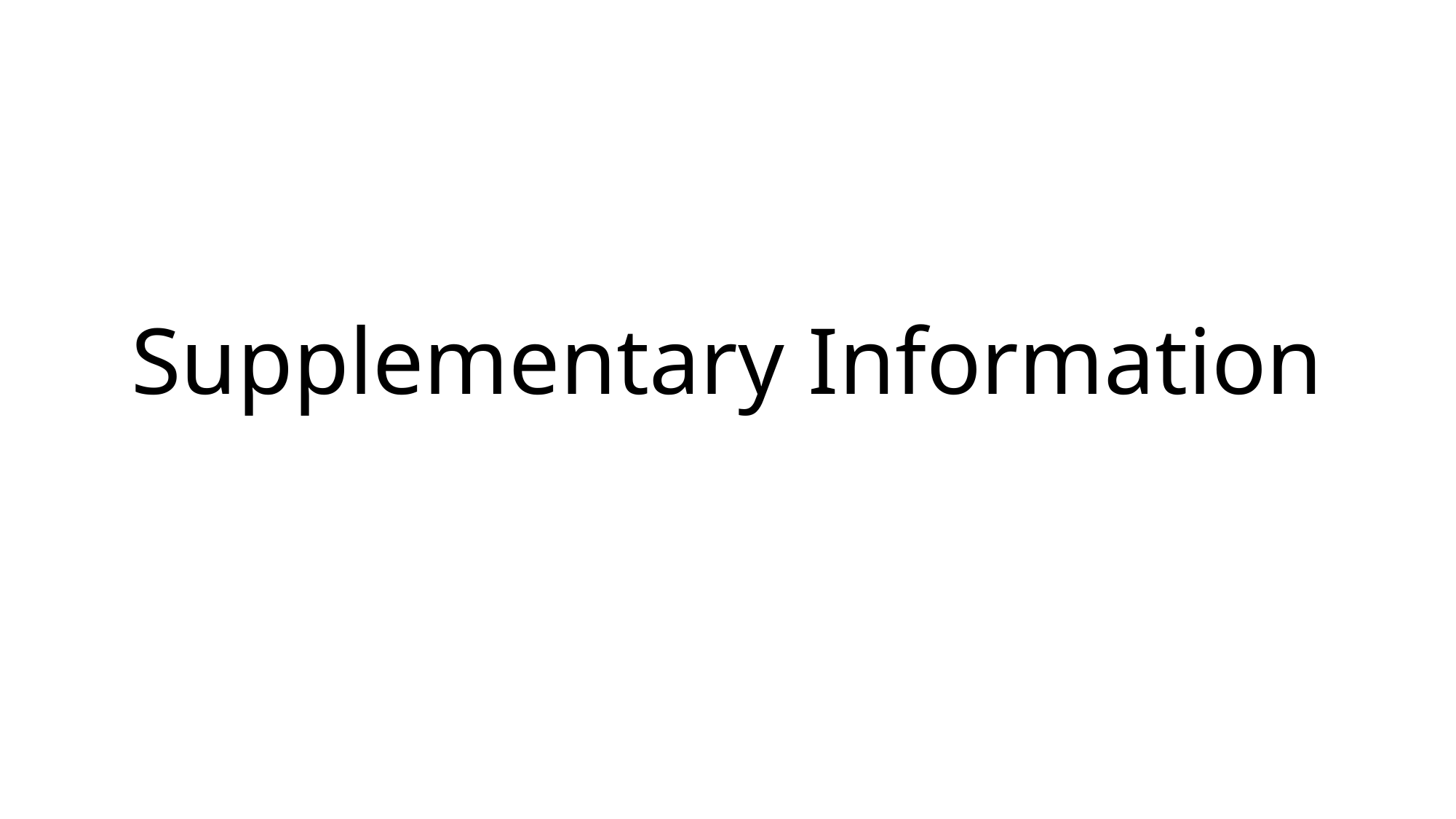

# Supplementary Information

## Slide 2
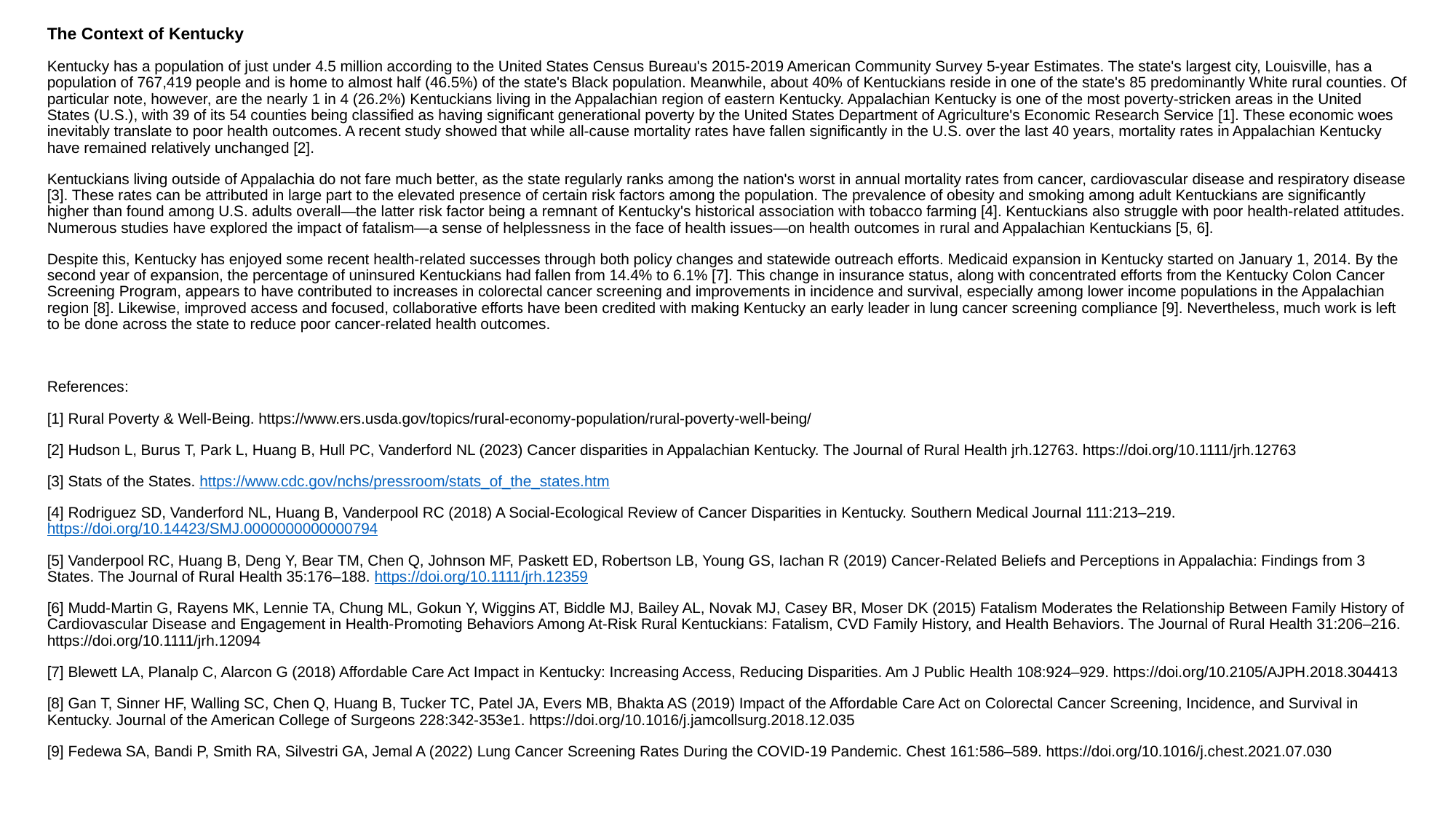

The Context of Kentucky
Kentucky has a population of just under 4.5 million according to the United States Census Bureau's 2015-2019 American Community Survey 5-year Estimates. The state's largest city, Louisville, has a population of 767,419 people and is home to almost half (46.5%) of the state's Black population. Meanwhile, about 40% of Kentuckians reside in one of the state's 85 predominantly White rural counties. Of particular note, however, are the nearly 1 in 4 (26.2%) Kentuckians living in the Appalachian region of eastern Kentucky. Appalachian Kentucky is one of the most poverty-stricken areas in the United States (U.S.), with 39 of its 54 counties being classified as having significant generational poverty by the United States Department of Agriculture's Economic Research Service [1]. These economic woes inevitably translate to poor health outcomes. A recent study showed that while all-cause mortality rates have fallen significantly in the U.S. over the last 40 years, mortality rates in Appalachian Kentucky have remained relatively unchanged [2].
Kentuckians living outside of Appalachia do not fare much better, as the state regularly ranks among the nation's worst in annual mortality rates from cancer, cardiovascular disease and respiratory disease [3]. These rates can be attributed in large part to the elevated presence of certain risk factors among the population. The prevalence of obesity and smoking among adult Kentuckians are significantly higher than found among U.S. adults overall—the latter risk factor being a remnant of Kentucky's historical association with tobacco farming [4]. Kentuckians also struggle with poor health-related attitudes. Numerous studies have explored the impact of fatalism—a sense of helplessness in the face of health issues—on health outcomes in rural and Appalachian Kentuckians [5, 6].
Despite this, Kentucky has enjoyed some recent health-related successes through both policy changes and statewide outreach efforts. Medicaid expansion in Kentucky started on January 1, 2014. By the second year of expansion, the percentage of uninsured Kentuckians had fallen from 14.4% to 6.1% [7]. This change in insurance status, along with concentrated efforts from the Kentucky Colon Cancer Screening Program, appears to have contributed to increases in colorectal cancer screening and improvements in incidence and survival, especially among lower income populations in the Appalachian region [8]. Likewise, improved access and focused, collaborative efforts have been credited with making Kentucky an early leader in lung cancer screening compliance [9]. Nevertheless, much work is left to be done across the state to reduce poor cancer-related health outcomes.
References:
[1] Rural Poverty & Well-Being. https://www.ers.usda.gov/topics/rural-economy-population/rural-poverty-well-being/
[2] Hudson L, Burus T, Park L, Huang B, Hull PC, Vanderford NL (2023) Cancer disparities in Appalachian Kentucky. The Journal of Rural Health jrh.12763. https://doi.org/10.1111/jrh.12763
[3] Stats of the States. https://www.cdc.gov/nchs/pressroom/stats_of_the_states.htm
[4] Rodriguez SD, Vanderford NL, Huang B, Vanderpool RC (2018) A Social-Ecological Review of Cancer Disparities in Kentucky. Southern Medical Journal 111:213–219. https://doi.org/10.14423/SMJ.0000000000000794
[5] Vanderpool RC, Huang B, Deng Y, Bear TM, Chen Q, Johnson MF, Paskett ED, Robertson LB, Young GS, Iachan R (2019) Cancer‐Related Beliefs and Perceptions in Appalachia: Findings from 3 States. The Journal of Rural Health 35:176–188. https://doi.org/10.1111/jrh.12359
[6] Mudd-Martin G, Rayens MK, Lennie TA, Chung ML, Gokun Y, Wiggins AT, Biddle MJ, Bailey AL, Novak MJ, Casey BR, Moser DK (2015) Fatalism Moderates the Relationship Between Family History of Cardiovascular Disease and Engagement in Health-Promoting Behaviors Among At-Risk Rural Kentuckians: Fatalism, CVD Family History, and Health Behaviors. The Journal of Rural Health 31:206–216. https://doi.org/10.1111/jrh.12094
[7] Blewett LA, Planalp C, Alarcon G (2018) Affordable Care Act Impact in Kentucky: Increasing Access, Reducing Disparities. Am J Public Health 108:924–929. https://doi.org/10.2105/AJPH.2018.304413
[8] Gan T, Sinner HF, Walling SC, Chen Q, Huang B, Tucker TC, Patel JA, Evers MB, Bhakta AS (2019) Impact of the Affordable Care Act on Colorectal Cancer Screening, Incidence, and Survival in Kentucky. Journal of the American College of Surgeons 228:342-353e1. https://doi.org/10.1016/j.jamcollsurg.2018.12.035
[9] Fedewa SA, Bandi P, Smith RA, Silvestri GA, Jemal A (2022) Lung Cancer Screening Rates During the COVID-19 Pandemic. Chest 161:586–589. https://doi.org/10.1016/j.chest.2021.07.030

## Slide 3
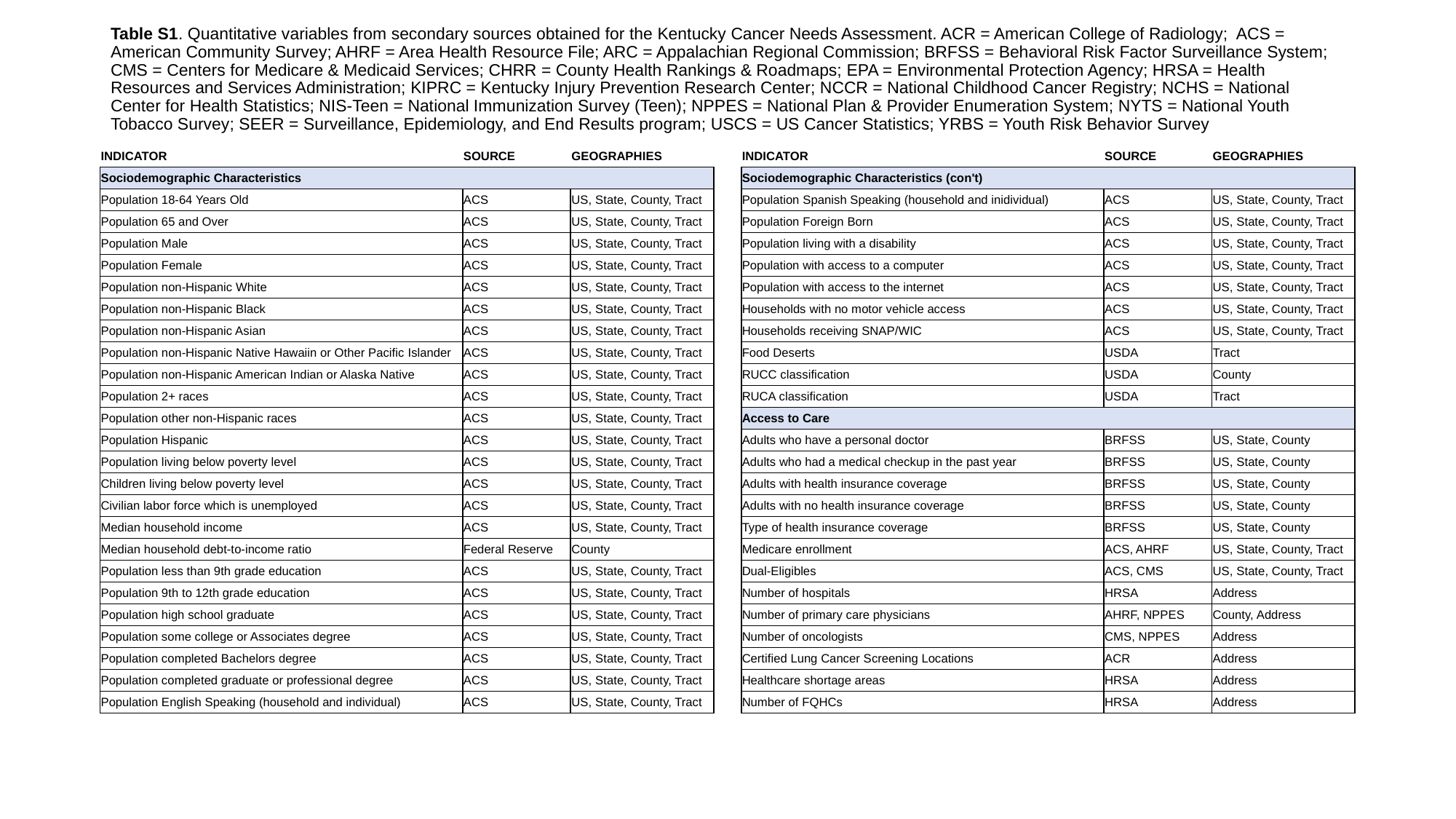

Table S1. Quantitative variables from secondary sources obtained for the Kentucky Cancer Needs Assessment. ACR = American College of Radiology; ACS = American Community Survey; AHRF = Area Health Resource File; ARC = Appalachian Regional Commission; BRFSS = Behavioral Risk Factor Surveillance System; CMS = Centers for Medicare & Medicaid Services; CHRR = County Health Rankings & Roadmaps; EPA = Environmental Protection Agency; HRSA = Health Resources and Services Administration; KIPRC = Kentucky Injury Prevention Research Center; NCCR = National Childhood Cancer Registry; NCHS = National Center for Health Statistics; NIS-Teen = National Immunization Survey (Teen); NPPES = National Plan & Provider Enumeration System; NYTS = National Youth Tobacco Survey; SEER = Surveillance, Epidemiology, and End Results program; USCS = US Cancer Statistics; YRBS = Youth Risk Behavior Survey
| INDICATOR | SOURCE | GEOGRAPHIES | | INDICATOR | SOURCE | GEOGRAPHIES |
| --- | --- | --- | --- | --- | --- | --- |
| Sociodemographic Characteristics | | | | Sociodemographic Characteristics (con't) | | |
| Population 18-64 Years Old | ACS | US, State, County, Tract | | Population Spanish Speaking (household and inidividual) | ACS | US, State, County, Tract |
| Population 65 and Over | ACS | US, State, County, Tract | | Population Foreign Born | ACS | US, State, County, Tract |
| Population Male | ACS | US, State, County, Tract | | Population living with a disability | ACS | US, State, County, Tract |
| Population Female | ACS | US, State, County, Tract | | Population with access to a computer | ACS | US, State, County, Tract |
| Population non-Hispanic White | ACS | US, State, County, Tract | | Population with access to the internet | ACS | US, State, County, Tract |
| Population non-Hispanic Black | ACS | US, State, County, Tract | | Households with no motor vehicle access | ACS | US, State, County, Tract |
| Population non-Hispanic Asian | ACS | US, State, County, Tract | | Households receiving SNAP/WIC | ACS | US, State, County, Tract |
| Population non-Hispanic Native Hawaiin or Other Pacific Islander | ACS | US, State, County, Tract | | Food Deserts | USDA | Tract |
| Population non-Hispanic American Indian or Alaska Native | ACS | US, State, County, Tract | | RUCC classification | USDA | County |
| Population 2+ races | ACS | US, State, County, Tract | | RUCA classification | USDA | Tract |
| Population other non-Hispanic races | ACS | US, State, County, Tract | | Access to Care | | |
| Population Hispanic | ACS | US, State, County, Tract | | Adults who have a personal doctor | BRFSS | US, State, County |
| Population living below poverty level | ACS | US, State, County, Tract | | Adults who had a medical checkup in the past year | BRFSS | US, State, County |
| Children living below poverty level | ACS | US, State, County, Tract | | Adults with health insurance coverage | BRFSS | US, State, County |
| Civilian labor force which is unemployed | ACS | US, State, County, Tract | | Adults with no health insurance coverage | BRFSS | US, State, County |
| Median household income | ACS | US, State, County, Tract | | Type of health insurance coverage | BRFSS | US, State, County |
| Median household debt-to-income ratio | Federal Reserve | County | | Medicare enrollment | ACS, AHRF | US, State, County, Tract |
| Population less than 9th grade education | ACS | US, State, County, Tract | | Dual-Eligibles | ACS, CMS | US, State, County, Tract |
| Population 9th to 12th grade education | ACS | US, State, County, Tract | | Number of hospitals | HRSA | Address |
| Population high school graduate | ACS | US, State, County, Tract | | Number of primary care physicians | AHRF, NPPES | County, Address |
| Population some college or Associates degree | ACS | US, State, County, Tract | | Number of oncologists | CMS, NPPES | Address |
| Population completed Bachelors degree | ACS | US, State, County, Tract | | Certified Lung Cancer Screening Locations | ACR | Address |
| Population completed graduate or professional degree | ACS | US, State, County, Tract | | Healthcare shortage areas | HRSA | Address |
| Population English Speaking (household and individual) | ACS | US, State, County, Tract | | Number of FQHCs | HRSA | Address |

## Slide 4
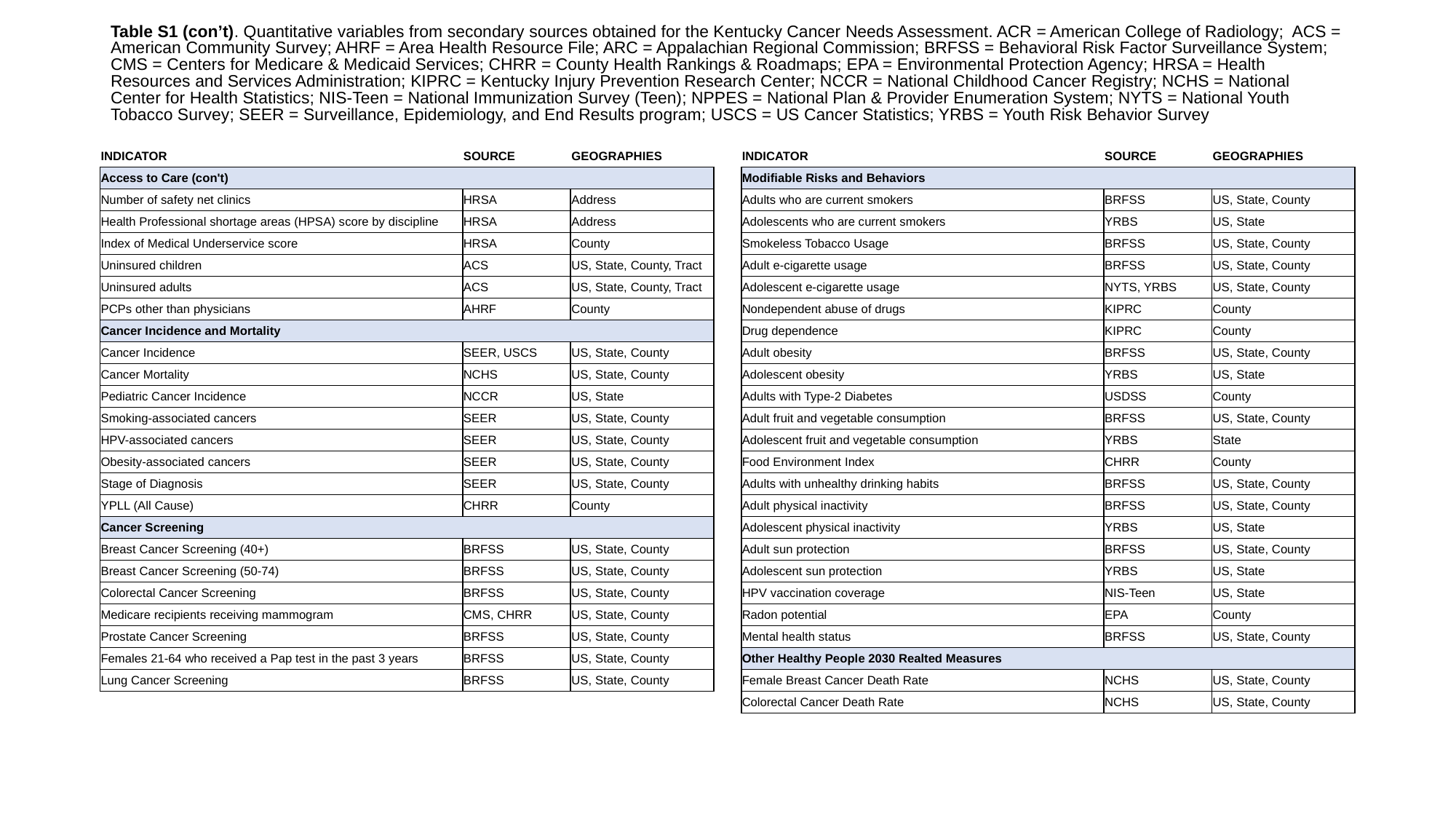

Table S1 (con’t). Quantitative variables from secondary sources obtained for the Kentucky Cancer Needs Assessment. ACR = American College of Radiology; ACS = American Community Survey; AHRF = Area Health Resource File; ARC = Appalachian Regional Commission; BRFSS = Behavioral Risk Factor Surveillance System; CMS = Centers for Medicare & Medicaid Services; CHRR = County Health Rankings & Roadmaps; EPA = Environmental Protection Agency; HRSA = Health Resources and Services Administration; KIPRC = Kentucky Injury Prevention Research Center; NCCR = National Childhood Cancer Registry; NCHS = National Center for Health Statistics; NIS-Teen = National Immunization Survey (Teen); NPPES = National Plan & Provider Enumeration System; NYTS = National Youth Tobacco Survey; SEER = Surveillance, Epidemiology, and End Results program; USCS = US Cancer Statistics; YRBS = Youth Risk Behavior Survey
| INDICATOR | SOURCE | GEOGRAPHIES | | INDICATOR | SOURCE | GEOGRAPHIES |
| --- | --- | --- | --- | --- | --- | --- |
| Access to Care (con't) | | | | Modifiable Risks and Behaviors | | |
| Number of safety net clinics | HRSA | Address | | Adults who are current smokers | BRFSS | US, State, County |
| Health Professional shortage areas (HPSA) score by discipline | HRSA | Address | | Adolescents who are current smokers | YRBS | US, State |
| Index of Medical Underservice score | HRSA | County | | Smokeless Tobacco Usage | BRFSS | US, State, County |
| Uninsured children | ACS | US, State, County, Tract | | Adult e-cigarette usage | BRFSS | US, State, County |
| Uninsured adults | ACS | US, State, County, Tract | | Adolescent e-cigarette usage | NYTS, YRBS | US, State, County |
| PCPs other than physicians | AHRF | County | | Nondependent abuse of drugs | KIPRC | County |
| Cancer Incidence and Mortality | | | | Drug dependence | KIPRC | County |
| Cancer Incidence | SEER, USCS | US, State, County | | Adult obesity | BRFSS | US, State, County |
| Cancer Mortality | NCHS | US, State, County | | Adolescent obesity | YRBS | US, State |
| Pediatric Cancer Incidence | NCCR | US, State | | Adults with Type-2 Diabetes | USDSS | County |
| Smoking-associated cancers | SEER | US, State, County | | Adult fruit and vegetable consumption | BRFSS | US, State, County |
| HPV-associated cancers | SEER | US, State, County | | Adolescent fruit and vegetable consumption | YRBS | State |
| Obesity-associated cancers | SEER | US, State, County | | Food Environment Index | CHRR | County |
| Stage of Diagnosis | SEER | US, State, County | | Adults with unhealthy drinking habits | BRFSS | US, State, County |
| YPLL (All Cause) | CHRR | County | | Adult physical inactivity | BRFSS | US, State, County |
| Cancer Screening | | | | Adolescent physical inactivity | YRBS | US, State |
| Breast Cancer Screening (40+) | BRFSS | US, State, County | | Adult sun protection | BRFSS | US, State, County |
| Breast Cancer Screening (50-74) | BRFSS | US, State, County | | Adolescent sun protection | YRBS | US, State |
| Colorectal Cancer Screening | BRFSS | US, State, County | | HPV vaccination coverage | NIS-Teen | US, State |
| Medicare recipients receiving mammogram | CMS, CHRR | US, State, County | | Radon potential | EPA | County |
| Prostate Cancer Screening | BRFSS | US, State, County | | Mental health status | BRFSS | US, State, County |
| Females 21-64 who received a Pap test in the past 3 years | BRFSS | US, State, County | | Other Healthy People 2030 Realted Measures | | |
| Lung Cancer Screening | BRFSS | US, State, County | | Female Breast Cancer Death Rate | NCHS | US, State, County |
| | | | | Colorectal Cancer Death Rate | NCHS | US, State, County |

## Slide 5
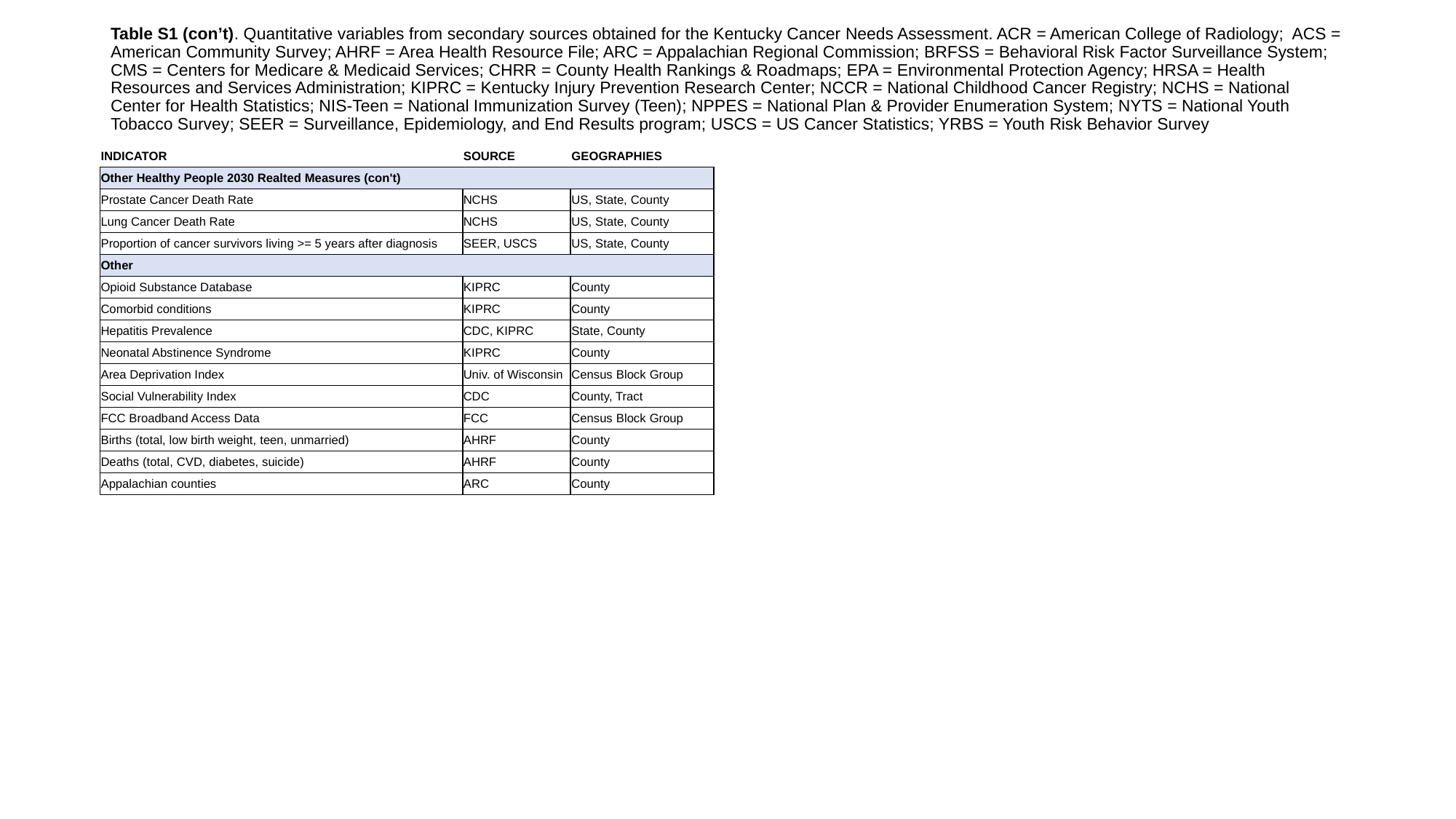

Table S1 (con’t). Quantitative variables from secondary sources obtained for the Kentucky Cancer Needs Assessment. ACR = American College of Radiology; ACS = American Community Survey; AHRF = Area Health Resource File; ARC = Appalachian Regional Commission; BRFSS = Behavioral Risk Factor Surveillance System; CMS = Centers for Medicare & Medicaid Services; CHRR = County Health Rankings & Roadmaps; EPA = Environmental Protection Agency; HRSA = Health Resources and Services Administration; KIPRC = Kentucky Injury Prevention Research Center; NCCR = National Childhood Cancer Registry; NCHS = National Center for Health Statistics; NIS-Teen = National Immunization Survey (Teen); NPPES = National Plan & Provider Enumeration System; NYTS = National Youth Tobacco Survey; SEER = Surveillance, Epidemiology, and End Results program; USCS = US Cancer Statistics; YRBS = Youth Risk Behavior Survey
| INDICATOR | SOURCE | GEOGRAPHIES | | | | |
| --- | --- | --- | --- | --- | --- | --- |
| Other Healthy People 2030 Realted Measures (con't) | | | | | | |
| Prostate Cancer Death Rate | NCHS | US, State, County | | | | |
| Lung Cancer Death Rate | NCHS | US, State, County | | | | |
| Proportion of cancer survivors living >= 5 years after diagnosis | SEER, USCS | US, State, County | | | | |
| Other | | | | | | |
| Opioid Substance Database | KIPRC | County | | | | |
| Comorbid conditions | KIPRC | County | | | | |
| Hepatitis Prevalence | CDC, KIPRC | State, County | | | | |
| Neonatal Abstinence Syndrome | KIPRC | County | | | | |
| Area Deprivation Index | Univ. of Wisconsin | Census Block Group | | | | |
| Social Vulnerability Index | CDC | County, Tract | | | | |
| FCC Broadband Access Data | FCC | Census Block Group | | | | |
| Births (total, low birth weight, teen, unmarried) | AHRF | County | | | | |
| Deaths (total, CVD, diabetes, suicide) | AHRF | County | | | | |
| Appalachian counties | ARC | County | | | | |

## Slide 6
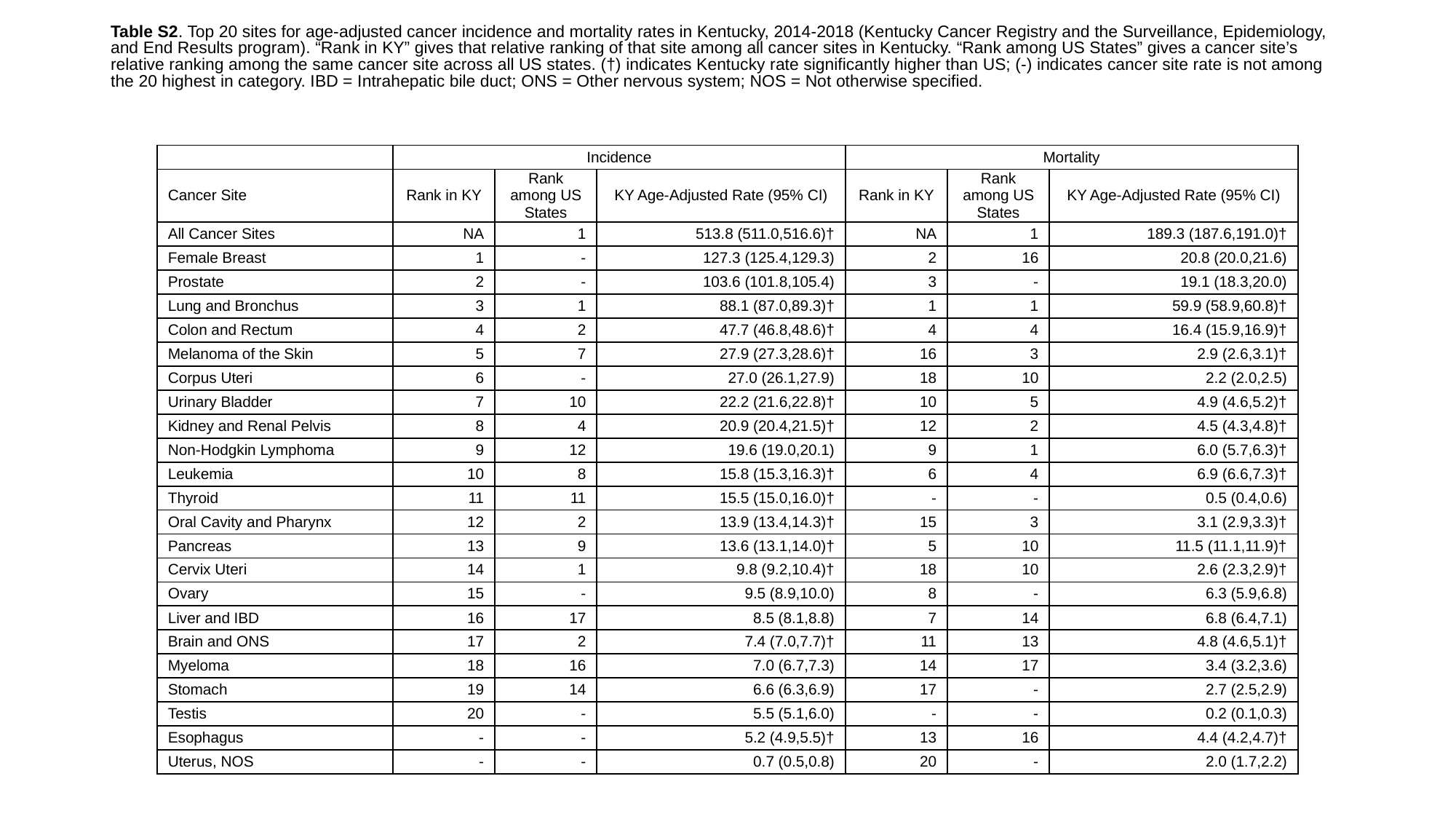

Table S2. Top 20 sites for age-adjusted cancer incidence and mortality rates in Kentucky, 2014-2018 (Kentucky Cancer Registry and the Surveillance, Epidemiology, and End Results program). “Rank in KY” gives that relative ranking of that site among all cancer sites in Kentucky. “Rank among US States” gives a cancer site’s relative ranking among the same cancer site across all US states. (†) indicates Kentucky rate significantly higher than US; (-) indicates cancer site rate is not among the 20 highest in category. IBD = Intrahepatic bile duct; ONS = Other nervous system; NOS = Not otherwise specified.
| | Incidence | | | Mortality | | |
| --- | --- | --- | --- | --- | --- | --- |
| Cancer Site | Rank in KY | Rank among US States | KY Age-Adjusted Rate (95% CI) | Rank in KY | Rank among US States | KY Age-Adjusted Rate (95% CI) |
| All Cancer Sites | NA | 1 | 513.8 (511.0,516.6)† | NA | 1 | 189.3 (187.6,191.0)† |
| Female Breast | 1 | - | 127.3 (125.4,129.3) | 2 | 16 | 20.8 (20.0,21.6) |
| Prostate | 2 | - | 103.6 (101.8,105.4) | 3 | - | 19.1 (18.3,20.0) |
| Lung and Bronchus | 3 | 1 | 88.1 (87.0,89.3)† | 1 | 1 | 59.9 (58.9,60.8)† |
| Colon and Rectum | 4 | 2 | 47.7 (46.8,48.6)† | 4 | 4 | 16.4 (15.9,16.9)† |
| Melanoma of the Skin | 5 | 7 | 27.9 (27.3,28.6)† | 16 | 3 | 2.9 (2.6,3.1)† |
| Corpus Uteri | 6 | - | 27.0 (26.1,27.9) | 18 | 10 | 2.2 (2.0,2.5) |
| Urinary Bladder | 7 | 10 | 22.2 (21.6,22.8)† | 10 | 5 | 4.9 (4.6,5.2)† |
| Kidney and Renal Pelvis | 8 | 4 | 20.9 (20.4,21.5)† | 12 | 2 | 4.5 (4.3,4.8)† |
| Non-Hodgkin Lymphoma | 9 | 12 | 19.6 (19.0,20.1) | 9 | 1 | 6.0 (5.7,6.3)† |
| Leukemia | 10 | 8 | 15.8 (15.3,16.3)† | 6 | 4 | 6.9 (6.6,7.3)† |
| Thyroid | 11 | 11 | 15.5 (15.0,16.0)† | - | - | 0.5 (0.4,0.6) |
| Oral Cavity and Pharynx | 12 | 2 | 13.9 (13.4,14.3)† | 15 | 3 | 3.1 (2.9,3.3)† |
| Pancreas | 13 | 9 | 13.6 (13.1,14.0)† | 5 | 10 | 11.5 (11.1,11.9)† |
| Cervix Uteri | 14 | 1 | 9.8 (9.2,10.4)† | 18 | 10 | 2.6 (2.3,2.9)† |
| Ovary | 15 | - | 9.5 (8.9,10.0) | 8 | - | 6.3 (5.9,6.8) |
| Liver and IBD | 16 | 17 | 8.5 (8.1,8.8) | 7 | 14 | 6.8 (6.4,7.1) |
| Brain and ONS | 17 | 2 | 7.4 (7.0,7.7)† | 11 | 13 | 4.8 (4.6,5.1)† |
| Myeloma | 18 | 16 | 7.0 (6.7,7.3) | 14 | 17 | 3.4 (3.2,3.6) |
| Stomach | 19 | 14 | 6.6 (6.3,6.9) | 17 | - | 2.7 (2.5,2.9) |
| Testis | 20 | - | 5.5 (5.1,6.0) | - | - | 0.2 (0.1,0.3) |
| Esophagus | - | - | 5.2 (4.9,5.5)† | 13 | 16 | 4.4 (4.2,4.7)† |
| Uterus, NOS | - | - | 0.7 (0.5,0.8) | 20 | - | 2.0 (1.7,2.2) |

## Slide 7
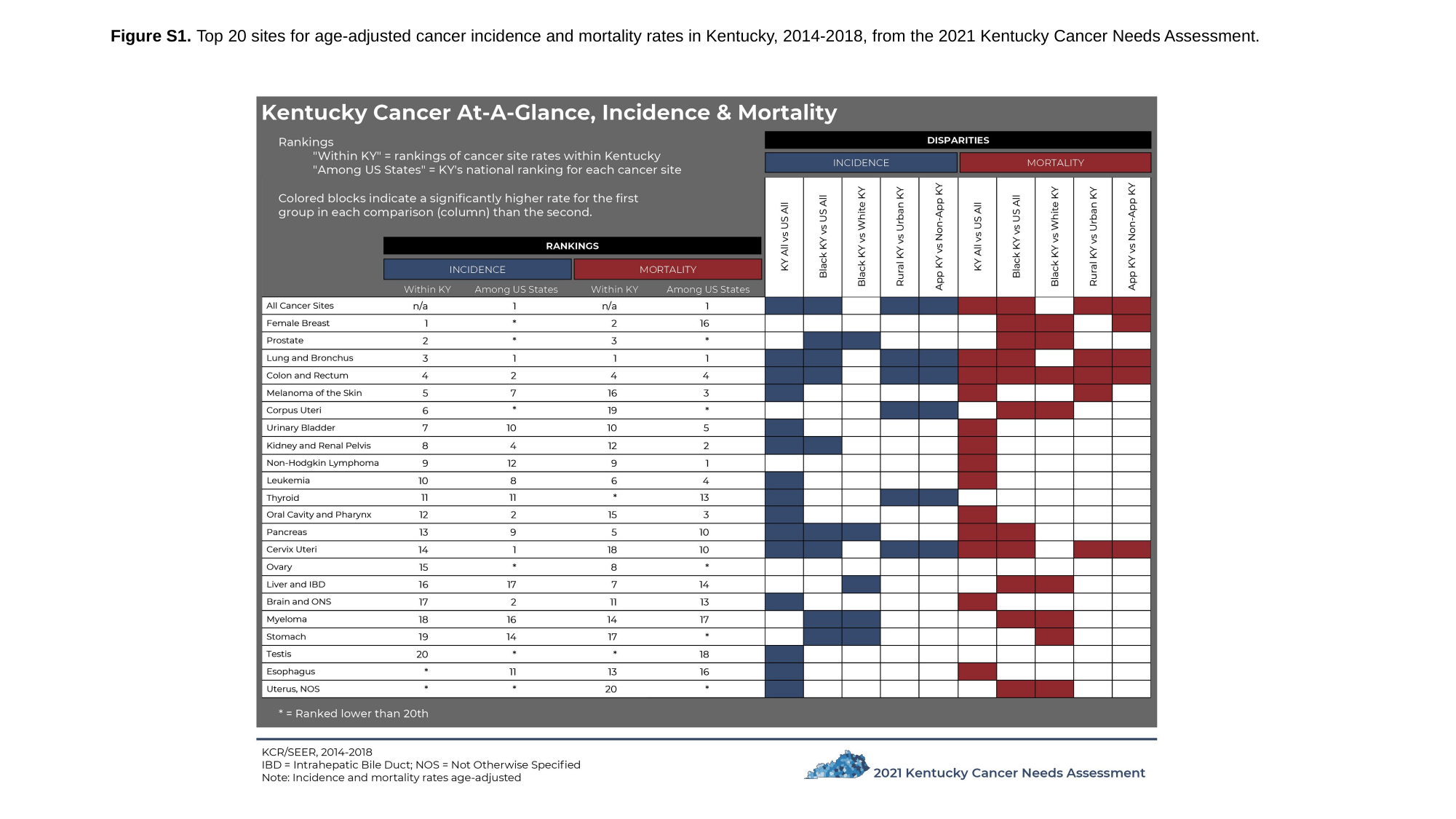

Figure S1. Top 20 sites for age-adjusted cancer incidence and mortality rates in Kentucky, 2014-2018, from the 2021 Kentucky Cancer Needs Assessment.

## Slide 8
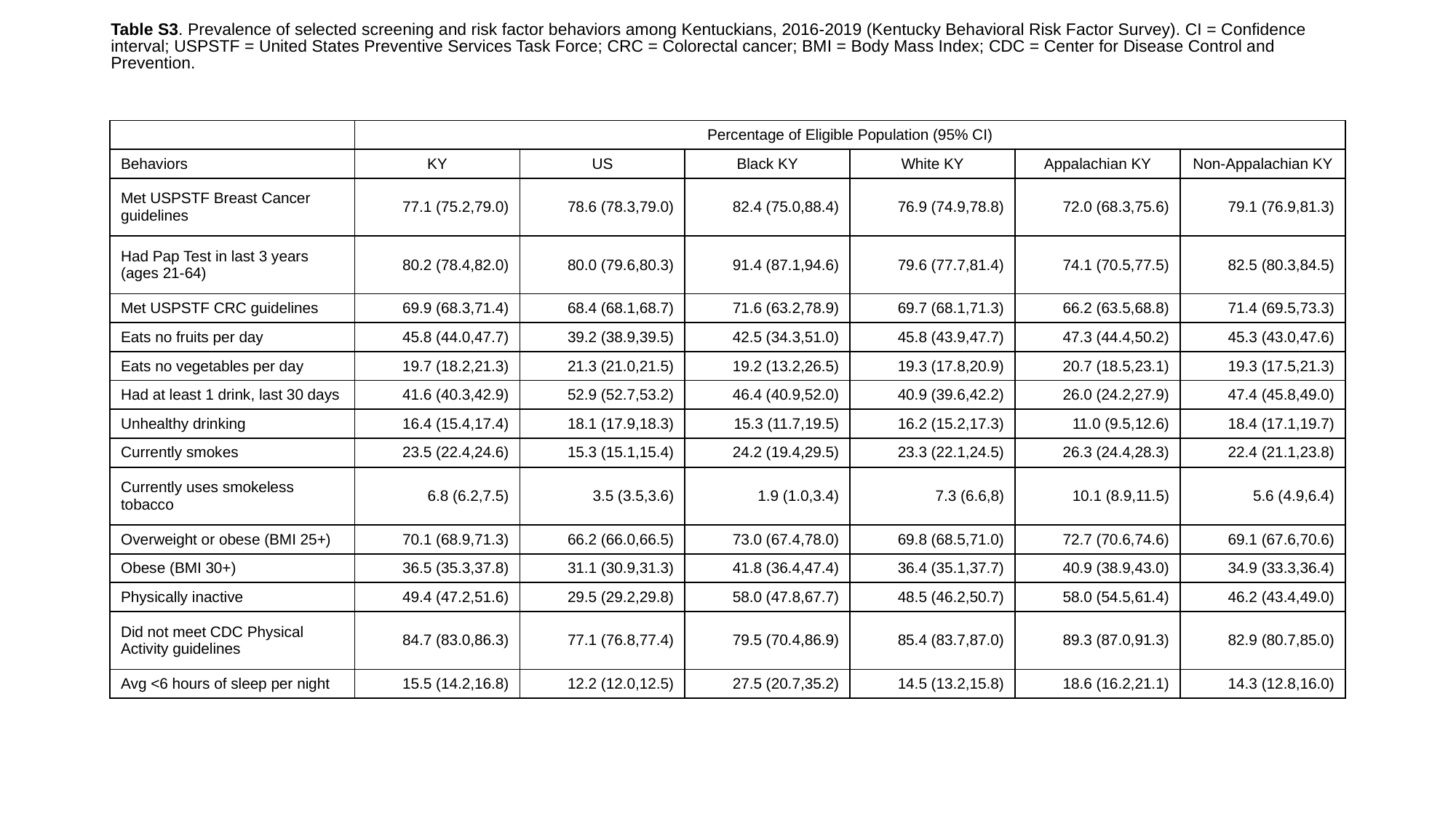

# Table S3. Prevalence of selected screening and risk factor behaviors among Kentuckians, 2016-2019 (Kentucky Behavioral Risk Factor Survey). CI = Confidence interval; USPSTF = United States Preventive Services Task Force; CRC = Colorectal cancer; BMI = Body Mass Index; CDC = Center for Disease Control and Prevention.
| | Percentage of Eligible Population (95% CI) | | | | | |
| --- | --- | --- | --- | --- | --- | --- |
| Behaviors | KY | US | Black KY | White KY | Appalachian KY | Non-Appalachian KY |
| Met USPSTF Breast Cancer guidelines | 77.1 (75.2,79.0) | 78.6 (78.3,79.0) | 82.4 (75.0,88.4) | 76.9 (74.9,78.8) | 72.0 (68.3,75.6) | 79.1 (76.9,81.3) |
| Had Pap Test in last 3 years (ages 21-64) | 80.2 (78.4,82.0) | 80.0 (79.6,80.3) | 91.4 (87.1,94.6) | 79.6 (77.7,81.4) | 74.1 (70.5,77.5) | 82.5 (80.3,84.5) |
| Met USPSTF CRC guidelines | 69.9 (68.3,71.4) | 68.4 (68.1,68.7) | 71.6 (63.2,78.9) | 69.7 (68.1,71.3) | 66.2 (63.5,68.8) | 71.4 (69.5,73.3) |
| Eats no fruits per day | 45.8 (44.0,47.7) | 39.2 (38.9,39.5) | 42.5 (34.3,51.0) | 45.8 (43.9,47.7) | 47.3 (44.4,50.2) | 45.3 (43.0,47.6) |
| Eats no vegetables per day | 19.7 (18.2,21.3) | 21.3 (21.0,21.5) | 19.2 (13.2,26.5) | 19.3 (17.8,20.9) | 20.7 (18.5,23.1) | 19.3 (17.5,21.3) |
| Had at least 1 drink, last 30 days | 41.6 (40.3,42.9) | 52.9 (52.7,53.2) | 46.4 (40.9,52.0) | 40.9 (39.6,42.2) | 26.0 (24.2,27.9) | 47.4 (45.8,49.0) |
| Unhealthy drinking | 16.4 (15.4,17.4) | 18.1 (17.9,18.3) | 15.3 (11.7,19.5) | 16.2 (15.2,17.3) | 11.0 (9.5,12.6) | 18.4 (17.1,19.7) |
| Currently smokes | 23.5 (22.4,24.6) | 15.3 (15.1,15.4) | 24.2 (19.4,29.5) | 23.3 (22.1,24.5) | 26.3 (24.4,28.3) | 22.4 (21.1,23.8) |
| Currently uses smokeless tobacco | 6.8 (6.2,7.5) | 3.5 (3.5,3.6) | 1.9 (1.0,3.4) | 7.3 (6.6,8) | 10.1 (8.9,11.5) | 5.6 (4.9,6.4) |
| Overweight or obese (BMI 25+) | 70.1 (68.9,71.3) | 66.2 (66.0,66.5) | 73.0 (67.4,78.0) | 69.8 (68.5,71.0) | 72.7 (70.6,74.6) | 69.1 (67.6,70.6) |
| Obese (BMI 30+) | 36.5 (35.3,37.8) | 31.1 (30.9,31.3) | 41.8 (36.4,47.4) | 36.4 (35.1,37.7) | 40.9 (38.9,43.0) | 34.9 (33.3,36.4) |
| Physically inactive | 49.4 (47.2,51.6) | 29.5 (29.2,29.8) | 58.0 (47.8,67.7) | 48.5 (46.2,50.7) | 58.0 (54.5,61.4) | 46.2 (43.4,49.0) |
| Did not meet CDC Physical Activity guidelines | 84.7 (83.0,86.3) | 77.1 (76.8,77.4) | 79.5 (70.4,86.9) | 85.4 (83.7,87.0) | 89.3 (87.0,91.3) | 82.9 (80.7,85.0) |
| Avg <6 hours of sleep per night | 15.5 (14.2,16.8) | 12.2 (12.0,12.5) | 27.5 (20.7,35.2) | 14.5 (13.2,15.8) | 18.6 (16.2,21.1) | 14.3 (12.8,16.0) |

## Slide 9
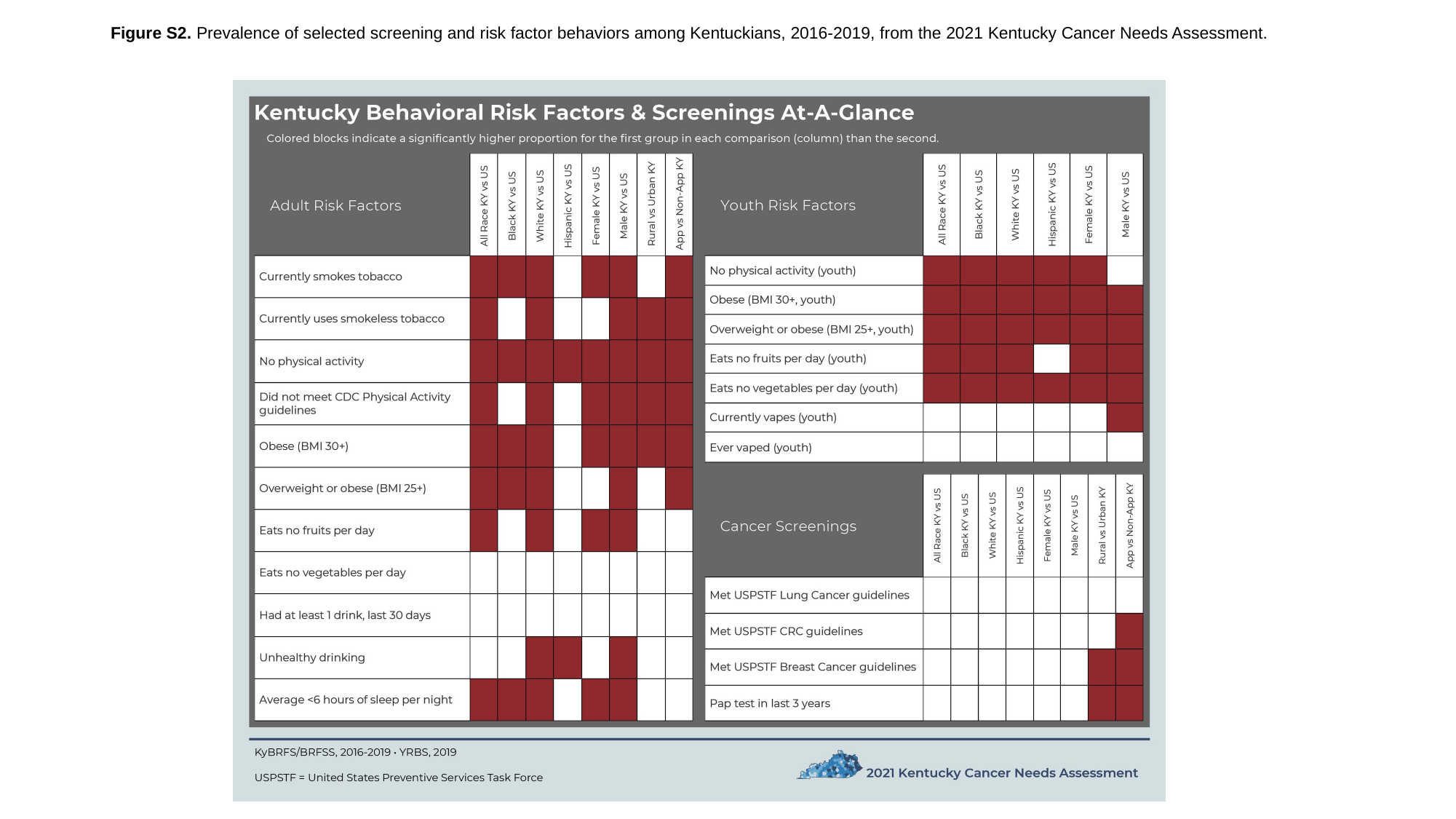

Figure S2. Prevalence of selected screening and risk factor behaviors among Kentuckians, 2016-2019, from the 2021 Kentucky Cancer Needs Assessment.
